# Micro‐Vessels‐Like 3D Scaffolds for Studying the Proton Radiobiology of Glioblastoma‐Endothelial Cells Co‐Culture Models

**DOI:** 10.1002/adhm.202302988

**Published:** 2023-11-27

**Authors:** Qais Akolawala, Floor Keuning, Marta Rovituso, Wouter van Burik, Ernst van der Wal, Henri H. Versteeg, Araci M. R. Rondon, Angelo Accardo

**Affiliations:** ^1^ Department of Precision and Microsystems Engineering Faculty of Mechanical Maritime and Materials Engineering Delft University of Technology Mekelweg 2 2628 CD Delft The Netherlands; ^2^ Holland Proton Therapy Center (HollandPTC) Huismansingel 4 2629 JH Delft The Netherlands; ^3^ Erasmus University College Nieuwemarkt 1A, Rotterdam 3011 HP Rotterdam The Netherlands; ^4^ Einthoven Laboratory for Vascular and Regenerative Medicine Division of Thrombosis and Hemostasis Department of Internal Medicine Leiden University Medical Center Albinusdreef 2 2333 ZA Leiden The Netherlands

**Keywords:** engineered cell microenvironments, glioblastoma, endothelial cells, proton therapy, two‐photon polymerization

## Abstract

Glioblastoma (GBM) is a devastating cancer of the brain with an extremely poor prognosis. While X‐ray radiotherapy and chemotherapy remain the current standard, proton beam therapy is an appealing alternative as protons can damage cancer cells while sparing the surrounding healthy tissue. However, the effects of protons on in vitro GBM models at the cellular level, especially when co‐cultured with endothelial cells, the building blocks of brain micro‐vessels, are still unexplored. In this work, novel 3D‐engineered scaffolds inspired by the geometry of brain microvasculature are designed, where GBM cells cluster and proliferate. The architectures are fabricated by two‐photon polymerization (2PP), pre‐cultured with endothelial cells (HUVECs), and then cultured with a human GBM cell line (U251). The micro‐vessel structures enable GBM in vivo‐like morphologies, and the results show a higher DNA double‐strand breakage in GBM monoculture samples when compared to the U251/HUVECs co‐culture, with cells in 2D featuring a larger number of DNA damage foci when compared to cells in 3D. The discrepancy in terms of proton radiation response indicates a difference in the radioresistance of the GBM cells mediated by the presence of HUVECs and the possible induction of stemness features that contribute to radioresistance and improved DNA repair.

## Graphical abstract

Brain micro‐vessels‐like scaffolds are realized using two‐photon polymerization (2PP). After co‐culturing them with endothelial (HUVECs) and glioblastoma (GBM) cells, which show in‐vivo‐like morphologies, they are exposed to proton radiation. Higher DNA damage is observed in GBM monoculture when compared to GBM/HUVECs co‐culture, with cells grown on 2D surfaces featuring a larger amount of DNA damage foci than cells in 3D.

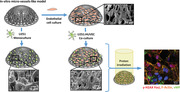

## 1 Introduction

Over the last two decades, there has been a rapid expansion of 3D cell culture models. The main reason for this is that 2D “Petri‐dish” approaches lead to the formation of unrealistic cell monolayers that do not reproduce the 3D spatial configuration assumed by cells in the in vivo environment. A 3D cell culture approach allows to recreate the specific environment that cells require in a controlled in vitro setting.^[^ ^1^, ^2^ ^]^ There are many additive manufacturing approaches to create polymeric or hydrogel “scaffolds”, which are structures that support cells in 3D.^[^ ^3^ ^]^ Among these methods, two‐photon polymerization (2PP) is a technique widely used in the cell mechanobiology field^[^ ^4^, ^5^ ^]^ since it can manufacture structures in the submicrometric range. The physical mechanism behind 2PP is two‐photon absorption (TPA) of near‐infrared radiation (NIR), which takes place by focusing femtosecond laser pulses onto an organic prepolymer material highly absorptive in the UV radiation range while “transparent” in the infrared radiation one. This nonlinear mechanism is tuned to induce the photopolymerization of the exposed material in extremely confined volumes called voxels.^[^ ^3^, ^6^ ^]^

Research and development of relevant models for the study of cancer cells is of paramount importance to study the fundamental aspects of cancer mechanobiology,^[^ ^7^, ^8^, ^9^, ^10^, ^11^ ^]^ as well as for prospective in vitro studies concerning treatments such as radiation and chemotherapy.^[^ ^12^, ^13^ ^]^ Among central nervous system (CNS) cancers, glioblastoma (GBM) is the most common. It accounts for 15% of all intracranial neoplasms and more than 50% of all gliomas.^[^ ^14^ ^]^ Glioblastoma is the most aggressive brain cancer and continues to have one of the most dismal prognoses of any cancer with a median survival rate of about 12–15 months and less than 5% of cases surviving more than 5 years after the original diagnosis.^[^ ^15^, ^16^, ^17^ ^]^ The current treatment approach for GBM involves surgical removal of the tumor followed by a combination of radiotherapy and chemotherapy. However, GBM often recurs, requiring multiple surgeries^[^ ^18^ ^]^ which are not always feasible, especially for tumors located in the deep core of the brain. Traditional X‐ray radiotherapy damages both cancerous and healthy brain tissue. A newer approach, proton beam therapy (PBT), uses protons^[^ ^19^ ^]^ instead of X‐rays, minimizing damage to healthy tissue. PBT's higher cost has limited its use^[^ ^20^, ^21^ ^]^ and comprehensive data on its effectiveness in treating GBM is lacking due to challenges in conducting cellular‐scale studies with animal models or biopsy tissues, due to scarcity issues and ethical reasons. Currently, the gold standard to study cancers, GBM in particular, involves intracranially injection of GBM cells into rodents^[^ ^22^ ^]^ and then euthanizing them to conduct histological analyses. This method is used for studying tumor progression and the response of tumor to chemotherapy.^[^ ^23^ ^]^ The limitations of this approach are the requirement of high‐precision cranial surgery, animal loss, and animal‐to‐animal variability.^[^ ^22^, ^24^, ^25^ ^]^ In order to overcome these limitations, it is, therefore, desirable to create reproducible 3D in vitro models, able to better mimic some of the geometric and biochemical properties of the GBM microenvironment compared to conventional 2D Petri‐dish monolayer cultures, which cannot reproduce the 3D spatial configuration followed by GBM cells in the real tumor.

Specifically, to study GBM in vitro, a bioinspired 3D model should ideally reproduce the brain micro‐vessel structures. This particular feature of the GBM microenvironment is of paramount importance because of the affinity that GBM cells have with nutrients and with micro‐vessels, where they cluster and proliferate.^[^ ^26^ ^]^ It is theorized that these nutrient‐rich regions allow the tumors to maintain a small amount of cells with stem‐like characteristics that contribute to GBM aggressiveness.^[^ ^27^, ^28^ ^]^ Medulloblastoma cells in this perivascular region showed no cell‐cycle arrest, or radiation‐induced cell death, indicating the importance of the region in the maintenance and regrowth of brain tumors.^[^ ^28^ ^]^ The study by Calabrese et al. demonstrated how rapidly cancer cells physically associate with endothelial cells even in an in vitro setting.^[^ ^27^ ^]^ They showed that endothelial cells regulate brain cancer by promoting the propagation and invasive potential of cancer cells in mice in vivo. They proposed that the brain tumor microvasculature contributes to the creation of microenvironments that promote the maintenance and formation of brain cancer‐associated stem cells. Calabrese et al. also demonstrated that increasing the number of endothelial cells in brain tumor xenografts leads to an increase in the self‐renewing of cancer stem cells and a higher rate of tumor expansion.^[^ ^27^ ^]^ Research conducted with different types of cancer, including glioma, indicates that endothelial cells can protect tumor cells from radiation‐induced damage,^[^ ^29^, ^30^, ^31^ ^]^ and the use of antibodies suppressing vascular endothelial growth factor (VEGF) associated with endothelial cell growth can sensitize even radioresistant GBM cell lines such as U87 to low radiation doses.^[^ ^32^ ^]^ Farin et al. in their work also showed that transplanted glioma cells migrate and proliferate using the host vasculature even in the absence of blood.^[^ ^26^ ^]^ Their dynamic experiments showed how the proliferation of cells occurred primarily at vascular branch points, suggesting that cell division is triggered by local environmental cues.

Endothelial cells thus play a crucial role in the GBM microenvironment. Along with contributing to GBM aggressiveness and radioresistance, endothelial cells also contribute structurally to the stability of the tumor.^[^ ^32^, ^33^ ^]^ The inclusion of the endothelial cells within in vitro GBM models is therefore an important parameter in the replication of the in vivo GBM environment. The use of biofidelic engineered GBM microenvironments enables the identifications of cell‐cell interactions^[^ ^11^ ^]^ that can be difficult to visualize in vivo looking at tumor masses, which, as mentioned earlier, suffer from subject‐to‐subject reproducibility.^[^ ^22^, ^24^ ^]^ An advantage of biomimetic 3D in vitro models is the possibility of studying patient‐specific responses. In a disease such as GBM where there is very high variability between patients and heterogeneity within tumor cells, a patient‐specific model could prove useful in radiation and chemotherapy dose determination. Yi et al. demonstrated this application by bioprinting GBM in vitro models to study patient‐specific responses to chemoradiotherapy.^[^ ^34^ ^]^ They employed the co‐culture of the U87 GBM cell line and HUVECs as a test model, along with a bioprinted decellularized matrix. Their model replicated the brain extracellular matrix (ECM) by incorporating biochemical (i.e., cells and ECM components) and biophysical cues (i.e., the structure). These models have studied the importance of the interactions between GBM and endothelial cells, but lack the spatial resolution of the micro‐vessel architecture as observed in scanning electron microscopy studies of human brain vasculature.^[^ ^35^ ^]^ The reproduction of these micrometric environments is therefore of paramount importance to study physiologically relevant interactions of cells with each other and their environment.

The use of high‐resolution 3D printing methods such as two‐photon polymerization allows us to recreate the micrometric topography of 3D cell microenvironments. For example, Marino et al. reported an in vitro testing model for the blood‐brain barrier,^[^ ^13^ ^]^ and, more recently, they also integrated magnetic self‐assembly functionalities.^[^ ^36^ ^]^ In our previous work,^[^ ^12^ ^]^ we also employed two‐photon polymerization to create engineered GBM cell microenvironments, featuring simple 3D networks of straight polymer beams, which were then exposed to proton beam radiation and showed how GBM cells in 3D exhibit lower DNA double‐strand breaks compared to 2D GBM monolayers grown on the same material.

In addition to the overall scaffold geometry and the biochemical cues, also topography, roughness, stiffness, and curvature play a role in cell mechanobiology. Rectilinear geometries are less biofidelic than curved structures as curvature landscapes can guide mechanisms such as cell migration.^[^ ^37^, ^38^ ^]^ Sub‐micron features also affect cells directly because they have a size similar to the ECM proteins, while microscale features can foster cell‐cell interactions and signaling.^[^ ^38^, ^39^ ^]^ The main rationale behind the design of such cell scaffolds is the local ECM, which plays an important role in providing a biomechanical niche for tumor cells to proliferate and spread.^[^ ^40^, ^41^ ^]^ Finally, the use of locally asymmetric lattice networks of varying porosity assists as well the migration and proliferation of cells. Topotaxis (cell movement directed by topography) can account for varying responses of cancer cells and provide further context to the study of diseases such as GBM.^[^ ^42^, ^43^ ^]^

In this work, we report the creation of a standardized, reproducible, biomimetic micro‐vessels‐like 3D scaffold featuring curved geometries, fabricated by 2PP, to foster the co‐culture of GBM (U251) cells and human umbilical vein endothelial cells (HUVECs), and assess the DNA damage induced by proton beams to U251 cells in vitro, both in monoculture and co‐culture configuration. The model design draws inspiration from micrographs of brain microvasculature^[^ ^35^ ^]^ and recreates 3D micro‐vessels‐like architectures to study the GBM cells and their interactions with the environment at a cellular level in a proton radiobiology context. The scaffolds are fabricated using the biomaterial IP‐Visio, which has high biocompatibility, and negligible autofluorescence.^[^ ^12^ ^]^ The model is designed to compare the DNA damage response of U251/HUVECs co‐cultures and U251 monocultures after being exposed to a dose of 0 and 8 Gy of proton irradiation in the Spread‐Out Bragg‐Peak configuration (SOBP) both in IP‐Visio 3D scaffolds and on 2D IP‐Visio pedestal structures. In addition, we report a comparative morphological characterization of cells in 2D and 3D configurations using scanning electron microscopy and 3D immunofluorescence imaging. Besides lower DNA damage in 3D cell microenvironments compared to 2D ones, the GBM/HUVECs co‐culture 3D models also featured lower DNA damage when compared to the U251 monoculture models. This shows that the integration of biochemical cues, induced by the presence of endothelial cells, affects the response to proton radiation of GBM cells, and paves the way for a series of studies involving other cell types (e.g., microglia, pericytes),^[^ ^44^ ^]^ patient‐derived GB^[^ ^11^ ^]^ and alternative proton treatments such as FLASH^[^ ^45^ ^]^ (based on ultra‐fast dose rates) in presence of the developed 3D biomimetic design.

## 2 Results and Discussion

### 2.1 Design and Fabrication of 3D Micro‐Vessels‐Like Environments

The scaffold design is inspired by the geometry of the brain microvasculature. 3D engineered cell scaffolds mostly rely on simplified rectilinear designs to recreate the morphology of specific biological features, but the actual geometry of brain microvascular networks, as reported in SEM micrographs^[^ ^35^, ^46^ ^]^ and immunofluorescent imaging,^[^ ^26^ ^]^ is rather curved. The architecture, characteristically, features many vessels that overlap each other, and form a complex network with smooth curves and nodes from which the vessels branch out. The spaces in between these vessels and along their walls are where GBM cells cluster and proliferate.^[^ ^26^ ^]^ Glioma cells are also known to use these nutrient‐rich regions to migrate and proliferate extensively. Variations in the size of gaps between these vessels have also been reported^[^ ^47^ ^]^ and tumor‐associated vasculature features a median diameter range of 7–20 µm.^[^ ^48^ ^]^ For in vitro models, it has been shown that curvatures can affect the alignment and morphology of HUVECs and their intercellular interactions.^[^ ^49^ ^]^ The complex structures therefore have an effect on cell‐cell and cell‐matrix interactions, and the glioblastoma cells exploit such an environment with their high invasive capacity.^[^ ^50^ ^]^

**Figure 1:**
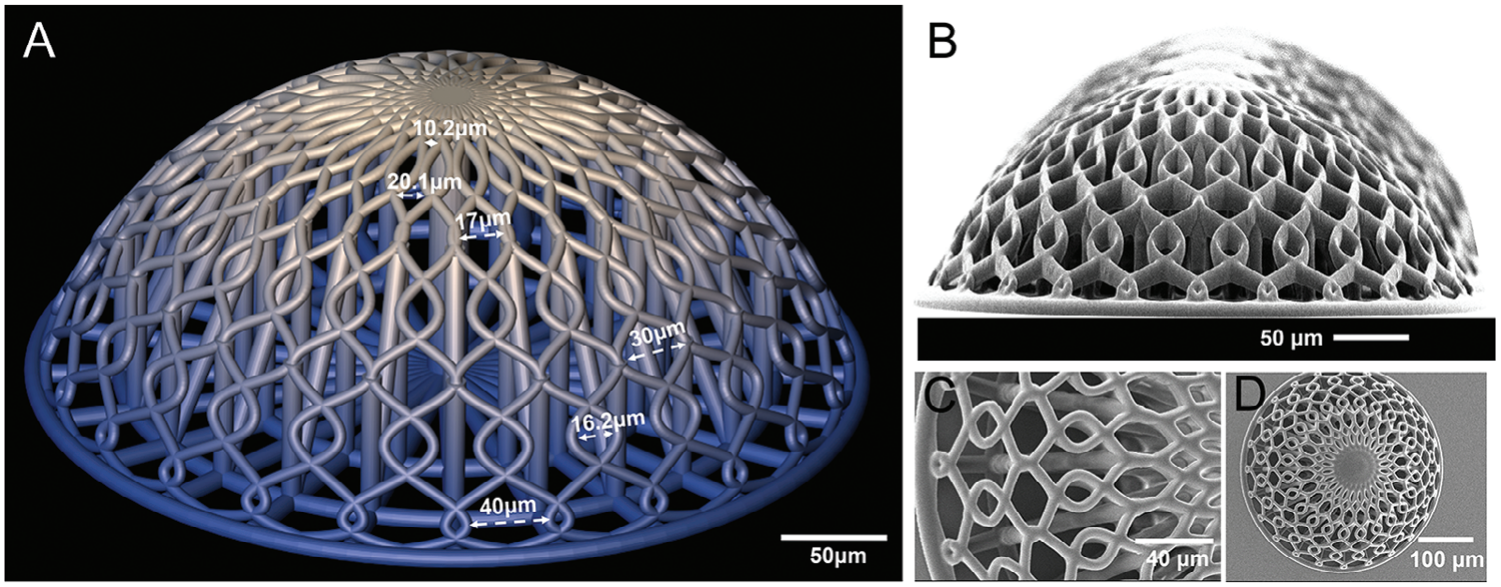
A) CAD design of the 3D scaffold with various gap sizes highlighted. B) SEM micrograph of the micro‐vessels‐like scaffolds at 90˚ degrees tilt. C) SEM micrograph close‐up of the scaffold's lattice structure from the top. D) SEM micrograph of the top view of a single scaffold.

In our 3D‐engineered micro‐vessels‐like model for GBM proton radiobiology, we included these design characteristics by using high‐resolution two‐photon polymerization to manufacture curved 3D features in a reproducible dome‐shaped lattice design as reported in **Figure** **1**. The overall height of the scaffolds is 140 µm with a base diameter of 380 µm. The largest pores of the lattice feature a gap of 40 µm which is comparable to what is observed in in vivo mice and human vasculature micrographs where they are ≈30 and 10–40 µm respectively.^[^ ^35^, ^46^ ^]^ These values are also in the range of the sizes of U251 cells (40–60 µm) and HUVECs (10–20 µm). The variation in the pore sizes of our scaffolds ranges from 10 to 40 µm and the unique shape of the lattice creates a heterogeneity in the gaps in the design.

This size range was also chosen to ensure the effective colonization of the scaffolds with both HUVECs and GBM cells. The differences in the sizes of the pores provide gradual variations in a controlled manner in an attempt to mimic the in vivo configuration.^[^ ^35^ ^]^ The lattice beams are 6 µm in diameter, with the dome being a section of a sphere, with a contact angle of 60°. To structurally support the weight of the dome lattice structure, several internal support pillars were required. 10 µm diameter internal pillars support the dome lattice, while 20 µm diameter central pillar provides stability and robustness to the design to also bear the loads and stresses of the cell culture process, changes in culture and rinsing solutions as well as dehydration steps for SEM characterization. These pillars can be seen in the sectional view of Figure S1, Supporting Information. Each sample set also includes three pedestals of 1000 × 1000 × 10 µm each as a control for cells growing on the same biomaterial but in flat 2D conditions (Figure S2, Supporting Information).

IP‐Visio was chosen because it is a material proven to have low autofluorescence and is not cytotoxic. The polymer did not require any biochemical functionalization to enable the adhesion and growth of GBM and HUVECs cells on the scaffolds. Although the value of Young's modulus of IP‐Visio^[^ ^12^ ^]^ is much higher than that of the brain ECM (0.1–1 kPa^[^ ^51^ ^]^), it is still about 53 times lower than that of conventional 2D cell culture substrates such as soda lime glass.^[^ ^12^ ^]^ ECM stiffness is shown to affect cell morphology, protein expression, and genetic expression.^[^ ^51^ ^]^ The lower Young's modulus combined with thin curved beam network geometry fosters cell‐cell and cell‐matrix interactions and induces differences in the cell morphologies compared to conventional glass substrates as is discussed further.

### 2.2 SEM and 3D Immunofluorescence Characterization of the U251 and HUVECs in Co‐cultures and Mono‐cultures

**Figure 2:**
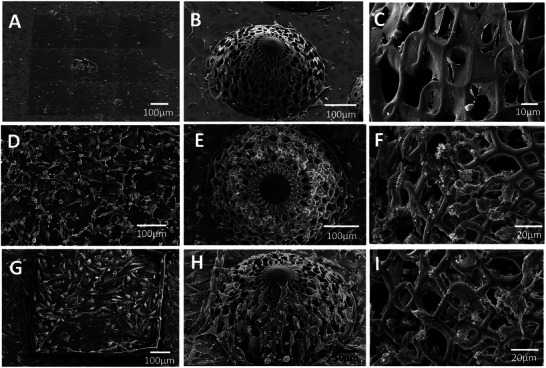
A) 2D IP‐Visio pedestals and B,C) micro‐vessels‐like scaffolds cultured with HUVECs only. D) 2D IP‐Visio pedestals and E,F) micro‐vessels‐like scaffolds cultured with U251 cells only. G) 2D IP‐Visio pedestals and H,I) micro‐vessels‐like scaffolds co‐cultured with U251 and HUVECs.

**Figure 3:**
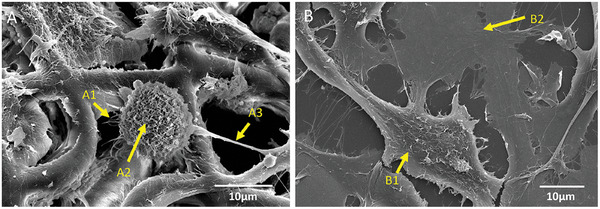
Detailed SEM micrographs of the U251/HUVEC Co‐culture. A) Co‐cultured cells on 3D micro‐vessels‐like structures ([A–1] indicates minor extensions of the cells, [A–2] indicates the microvilli on the surface of the cells, and [A–3] indicates the major extensions). B) Co‐cultured cells on 2D pedestals ([B–1] indicates U251 cells, [B–2] points at HUVECs).

Scanning electron microscopy and 3D immunofluorescence imaging were employed to assess the cell‐cell and cell‐matrix interactions of the HUVECs and U251 cells. **Figure** **2** shows representative SEM micrographs of the tested experimental conditions (2D/3D GBM and HUVECs monoculture, 2D/3D GBM/HUVECs co‐culture). The HUVECs are characterized by the formation of sheet‐like networks that are visible in the 3D micro‐vessels‐like scaffolds and the 2D pedestals (Figure 2A–C). In the 3D scaffolds, we also observed that the thin sheet‐like networks are free‐standing over the pores and that the cells wrap homogeneously the curved beams, which is very promising for further studies, where pericytes, playing a vital role in the blood‐brain barrier,^[^ ^52^ ^]^ could be included. Some low HUVECs cell density experiments (seeding density of 50 000 cells per mL) also show the longitudinal alignment of these cells along the micro‐vessels‐like 3D architecture (Figure S3B, Supporting Information). The U251 cells on the other hand are characterized by microvilli, observed on their outer surface,^[^ ^53^ ^]^ indicated in **Figure** **3**A (A2 arrow). The use of HMDS in the dehydration protocol allows to preserve such sub‐cellular structures (see Experimental Section). The HUVECs on the other hand are characterized by a smooth outer surface as indicated in Figures 2C and 3B (B2 arrow).^[^ ^54^ ^]^

U251 cells in 3D also show a more spherical morphology compared to GBM cells in 2D, which is in line with previous observations^[^ ^54^ ^]^ and better resembles the cellular morphology in vivo. The organization of the U251 cells in the co‐culture environments in 3D and 2D also present differences. The U251 cells showed an affinity for the corners formed by the micro‐vessels‐like geometry (Figures 2D–I and 3A). The cells in such a position also form extensions in multiple directions to exploit the geometry of the complex scaffolds. Such preferential adhesion of the GBM cells was desired to reproduce the physiological clustering taking place in real brain blood vessels.

**Figure 4:**
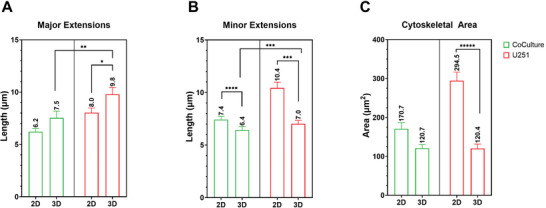
Quantification of major extensions’ length, minor extensions’ length, and cytoskeletal areas of U251 cells. The sample sizes (*n*) for each of the graphs are as follows: A) Co‐culture 2D = 27, co‐culture 3D = 42, U251 2D = 93, U251 3D = 37; B) Co‐culture 2D = 53, co‐culture 3D = 52, U251 2D = 49, U251 3D = 54; C) Co‐culture 2D = 21, co‐culture 3D = 16, U251 2D = 26, U251 3D = 23. The *p*‐values of the measurements are indicated. *p = 0.06, **p < 0.05, ***p < 0.01, ****p < 0.001, ***** *p* < 0.0001.

The U251 cells also feature the presence of minor (Figure 3A, A1 arrow) and major (Figure 3A, A3 arrow) extensions which are filopodia‐like membrane protrusions, that assist the cell in maneuvering, navigating, and invading its microenvironment.^[^ ^53^, ^55^ ^]^ The terms major and minor extensions were chosen here to distinguish between two main protrusion groups according to their physical characteristics as observed in the SEM micrographs. The major extensions have a diameter of 1–1.5 µm, while the minor extensions feature a “hair‐like” appearance with diameters of 0.3–0.5 µm. These morphological parameters have not been extensively investigated so far in the presence of 3D microenvironments.^[^ ^56^ ^]^ **Figure** **4** shows the quantification of three morphological features of the U251 cells in 2D/3D mono‐cultures/co‐cultures: major extensions, minor extensions (in 2D and 3D), and cytoskeletal area.

The major extensions of the GBM cells are longer in 3D as compared to 2D (both in mono‐ and co‐culture conditions, Figure 4A), owing to the ability of the cells to form larger networks over the 3D scaffolds by employing its curved beams as a guiding pathway. It is known indeed how glioma cells tend to move along the tracks provided by blood vessels.^[^ ^57^ ^]^ The scaffolds also allow for the formation of multidirectional processes in a 3D space and it is known how cancer cells are able to invade 3D environments by changing their shape.^[^ ^58^ ^]^ The minor extensions of the cells (i.e., the thinner filopodia) are, interestingly, longer for the 2D samples than for the 3D ones (Figure 4B). These structures are driven by complex cellular assemblies that are mainly studied in 2D.^[^ ^56^ ^]^ In presence of the 2D substrates, the thinner filopodia have larger surface areas to form longer connections. In contrast, these networks in 3D scaffolds need to bridge a larger overhanging space. It should also be noted that it is documented how cell processes of such dimensional range are more fragile and difficult to preserve for visualization.^[^ ^59^ ^]^ Figure 3A qualitatively shows that the minor extensions are also more numerous in 3D than in 2D. This could play a role in the context of radiation studies, as it has been shown that cells with greater migratory and invasive potential also show higher radioresistance.^[^ ^60^ ^]^ Figure 4C shows the much larger cytoskeletal areas in 2D as compared to 3D which is expected with GBM cells in 2D presenting a spread‐out morphology, when compared to the cells in 3D which show a more rounded morphology.^[^ ^54^ ^]^

**Figure 5:**
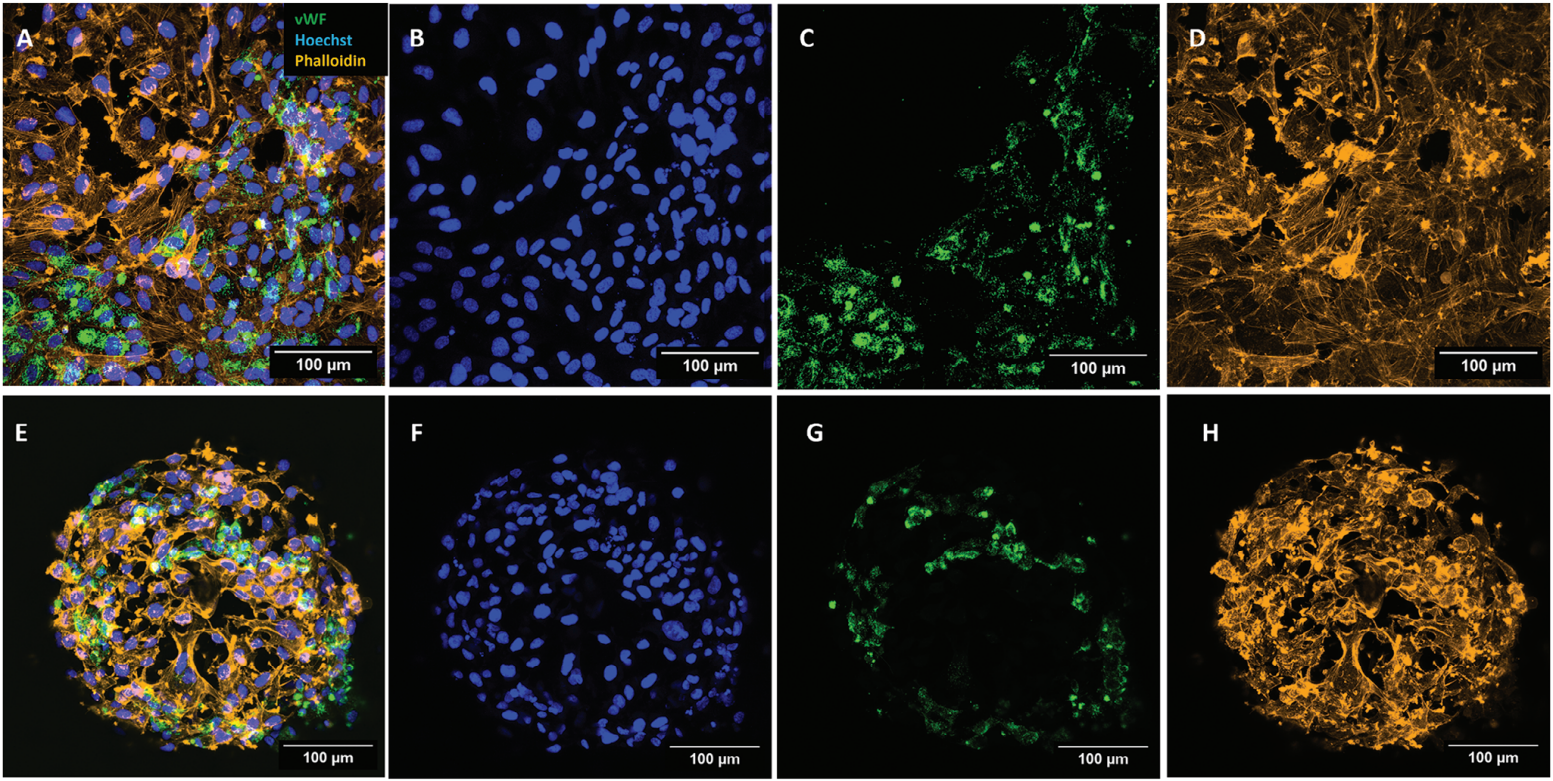
Confocal image *Z*‐projection for HUVECs and U251 cells co‐cultured on 2D pedestals and 3D scaffolds. A) Composite image showing Hoechst, Phalloidin, and vWF in 2D. B–D) images showing individual channels for a 2D pedestal. E) Composite image showing Hoechst, Phalloidin, and vWF in cells on a colonized 3D scaffold. F–H) Images showing individual channels for a 3D scaffold.

The immunofluorescence imaging of the co‐culture samples was carried out using Hoechst (blue) for the nuclei, Phalloidin‐TRITC conjugate (orange) for the cytoskeleton, and von‐Willebrand factor‐FITC conjugate (vWF, green), which is routinely employed for identifying blood vessels in tissue sections.^[^ ^61^ ^]^ vWF is used here to distinguish between GBM cells and HUVECs as Phalloidin and Hoechst stain both HUVECs and U251 cells^[^ ^61^, ^62^ ^]^ while vWF factor is a specific glycoprotein that is present only in endothelial cells and megakaryocytes. This protein resides in subcellular Weibel–Palade bodies that are unique to endothelial cells. The U251 cells do not express vWF, thus, this allowed us to distinguish between the two cell types in co‐culture. **Figure** **5** shows a representative image of the GBM/HUVECs co‐cultures on both 2D pedestals (Figure 5A–D) and 3D scaffolds (Figure 5E–H), with the composite and individual channel images. The green channel (5C and 5G) shows the vWF present expressed only by HUVECs. The cells that do not show vWF are U251 cells, which are clearly visualized in the composite images (Figures 5A and 5E).

### 2.3 Evaluation of DNA Damage of U251 Cell Networks and U251:HUVECs Co‐cultures in 3D‐Engineered Micro‐Vessels Environments and 2D Pedestals upon Proton Irradiation

Proton therapy is one of the most recent radiation treatment modalities. When compared to conventional X‐ray photon radiation, proton beams can be deposited in small, precise areas with minimal lateral scattering in tissue, ensuring that little to no radiation is delivered to healthy tissue surrounding the tumor.^[^ ^63^ ^]^ This makes proton therapy the preferred option for treating central nervous malignancies in order to minimize neurocognitive deficits in normal brain tissue.^[^ ^64^, ^65^ ^]^ Radiation induces DNA damage and cytotoxicity through direct DNA breaks and indirectly through the generation of reactive oxygen species (ROS). Compared to photons, protons are charged particles with greater mass and can produce a higher ionization density region which can generate more ROS than photon radiation.^[^ ^66^ ^]^

In the present work, we evaluated the DNA damage response of the U251 cells when exposed to a single dose (8 Gy) of proton beam radiation. Anti‐gamma H2A.X antibody is an established marker to quantify radiation‐induced DNA double‐strand breaks (DSBs) in cells.^[^ ^67^, ^68^, ^69^ ^]^ Gamma H2A.X foci formation is directly proportional to the extent of double‐strand DNA damage in a cell and is classified as a senescence biomarker.^[^ ^70^ ^]^ Figure S4 in the Supporting Information, shows the formation of the foci in GBM cells, on 2D pedestals, after exposure to 8 Gy proton radiation. Gamma H2A.X has already been specifically employed in the past for U251 cells. ^[^ ^71^, ^72^ ^]^ In our previous work we were able to establish that gamma H2A.X foci formation directly depended on the proton irradiation dose with cells exposed to a greater dose showing a higher amount of foci formation.^[^ ^12^ ^]^

**Figure 6:**
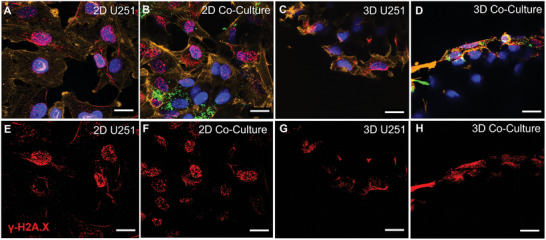
Confocal imaging of U251 monocultures and U251/HUVECs co‐cultures on 2D pedestals and 3D micro‐vessel‐like scaffolds upon 8 Gy SOBP proton radiation. A–D) Composite images of 2D pedestals and 3D scaffolds (Red: Gamma H2A.X Foci; Green: vWF‐FITC; Yellow: Phalloidin‐TRITC; Blue: Nuclei stained with Hoechst); E–H) Intranuclear Gamma H2A.X foci formation. Scale bars, 20 µm.

**Figure 7:**
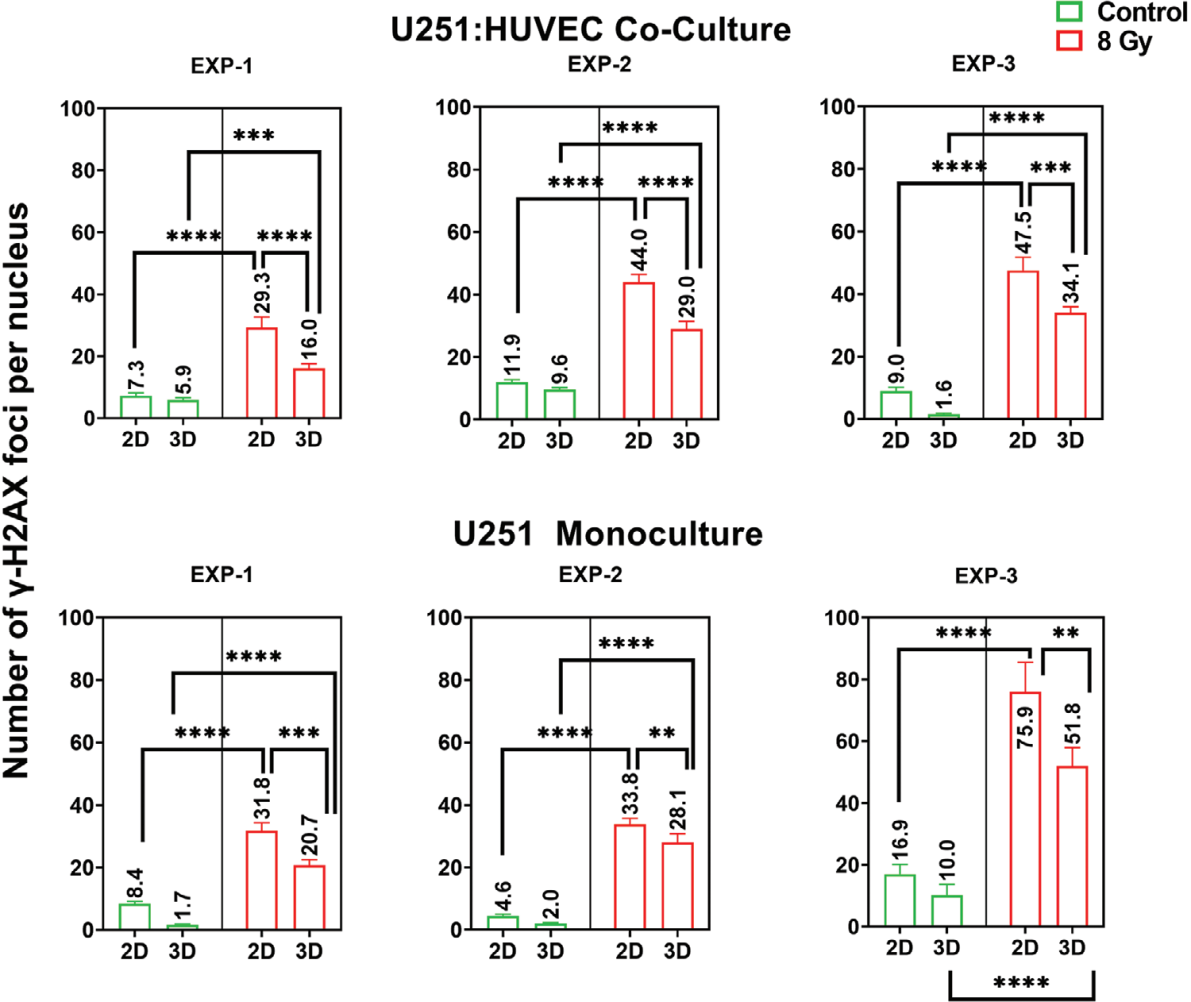
Quantification of the number of gamma H2A.X foci formed. Each graph shows the formation of gamma H2A.X foci for control and irradiated samples for different conditions. The trend of the data correlates across all three independent experiments. The charts show the mean and SD values. The *p*‐values of the measurements are indicated. *p < 0.05, **p < 0.01, ***p < 0.001, ****p < 0.0001. The sample size (*n*) for each condition is as follows: EXP‐1 control co‐culture 2D = 86, EXP‐1 control co‐culture 3D = 143, EXP‐1 irradiated co‐culture 2D = 82, EXP‐1 irradiated co‐culture 3D = 185; EXP‐2 control co‐culture 2D = 128, EXP‐2 control co‐culture 3D = 146, EXP‐2 irradiated co‐culture 2D = 116, EXP‐2 irradiated co‐culture 3D = 151; EXP‐3 control co‐culture 2D = 75, EXP‐3 control co‐culture 3D = 142, EXP‐3 irradiated co‐culture 2D = 87, EXP‐3 irradiated co‐culture 3D = 238. EXP‐1 control monoculture 2D = 234, EXP‐1 control monoculture 3D = 99, EXP‐1 irradiated monoculture 2D = 238, EXP‐1 irradiated monoculture 3D = 77; EXP‐2 control monoculture 2D = 182, EXP‐2 control monoculture 3D = 126, EXP‐2 irradiated monoculture 2D = 209, EXP‐2 irradiated monoculture 3D = 148. EXP‐3 control monoculture 2D = 40, EXP‐3 control monoculture 3D = 26, EXP‐3 irradiated monoculture 2D = 27, EXP‐3 irradiated monoculture 3D = 32.

The results of our previous work also showed that the DNA damage foci per cell are 10–20% higher (depending on the dose) on 2D pedestals when compared to simple rectilinear 3D scaffold counterparts.^[^ ^12^ ^]^ In the present work, we analysed the differences in terms of gamma H2A.X formation for i) U251 cells in monoculture and U251 cells in co‐culture with HUVECs in the presence of ii) 2D surfaces and 3D micro‐vessels‐like engineered environments. The foci counting strategy is described in the Experimental Section and Supporting Information (Figure S5, Supporting Information). As reported in **Figures** **6** and **7**, for all conditions, cells in 2D showed a larger number of DNA damage foci when compared to the cells in 3D, and a higher number of foci in irradiated samples when compared to the control (non‐irradiated) samples. The mechanical stimuli of the ECM are known to contribute to changes in cell sensing, signal transduction, cell apoptosis, and cell fate.^[^ ^73^, ^74^ ^]^ This result is in line with in vivo studies involving transplanted tumor cells in mice where GBM cells showed as well lower DNA damage than in vitro.^[^ ^75^ ^]^ The lower amount of DNA damage foci imply a higher radioresistance in the cells when compared to their 2D counterparts,^[^ ^76^, ^77^, ^78^ ^]^ but could also indicate a higher rate of repair mediated by the ECM.^[^ ^77^, ^79^ ^]^

**Figure 8:**
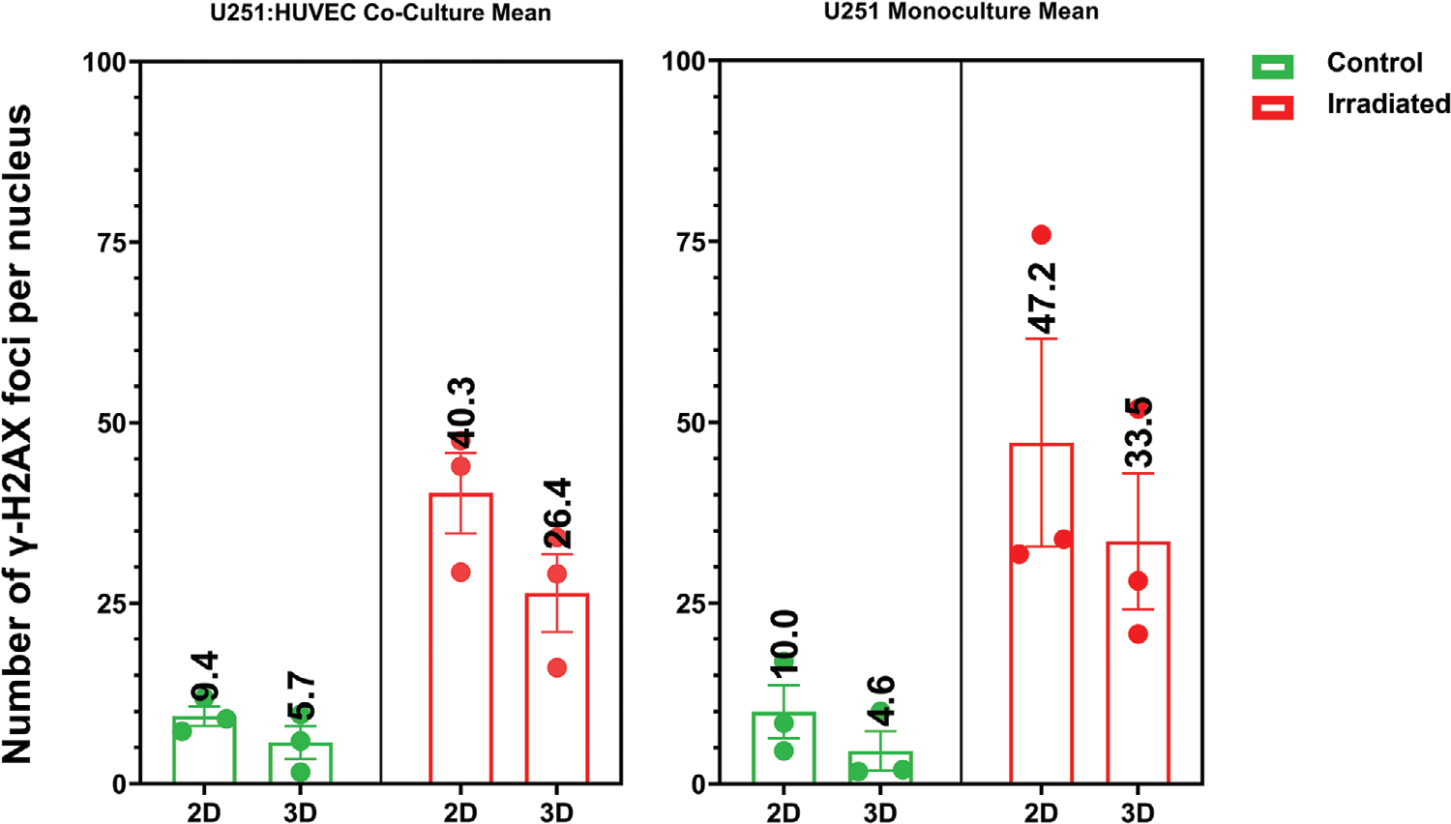
Mean values of the experiments reported in Figure 7. The mean values are higher for irradiated samples. The values for monoculture samples are ≈20% higher than for co‐culture samples.

The second observed result was that on average, the GBM cells in the U251/HUVEC co‐cultures performed on the 3D micro‐vessels‐like scaffolds showed fewer foci of DSB damage than those in the corresponding U251 monocultures (**Figure** **8**). It is important to note that DNA double‐strand breakage occurs for both U251 and HUVECs. The quantification of the data in Figure 7 only takes into account the DNA damage foci of the U251 cells. The experimental results show also on average a higher number of gamma H2A.X foci in the monoculture samples as compared to the co‐cultures, for cells in both 2D and 3D (Figure 8), with the monoculture samples showing 25% more foci in the cells in a 3D configuration and 20% more in GBM cells cultured on 2D pedestals, when compared to their respective co‐cultures.

These results suggest that all other parameters remaining the same, the presence of the engineered environments can influence the response of U251 cells to proton beam radiation. As stated previously, fewer gamma H2A.X foci imply higher radioresistance or altered repair kinetics mediated by the surrounding environment. In this context, we hypothesize that the presence of HUVECs contributed to the higher radioresistance. The HUVECs colonized the scaffold design following the micro‐vessels‐like architecture. The scaffold structures provide branching points where the U251 cells preferentially adhere and proliferate.^[^ ^26^ ^]^ This can also visually be observed in Figure 3. It has also been reported that cells growing in peri‐vascular niches can exhibit higher expression of stemness markers such as CD133.^[^ ^27^ ^]^ In the work of Nakod et al., it has been observed that 3D GBM/HUVEC co‐culture models show a higher expression of known stemness markers such as CD133, nestin, and SOX2.^[^ ^80^ ^]^ The presence of endothelial cells in co‐culture with the GBM cells produces a higher expression of these markers than the individual 3D monocultures.^[^ ^80^ ^]^ From these markers, CD133 is the most common marker used to sort glioma stem cells (GSCs).^[^ ^81^, ^82^ ^]^ Cells positive for CD133 have shown tight interactions with the vascular assembly that surrounds them and endothelial cell‐derived factors have been shown to accelerate the growth of brain tumors and cancer cell proliferation.^[^ ^27^ ^]^ Higher expression of CD133 in cells is linked to an increase in radioresistance and cell proliferation.^[^ ^78^, ^83^ ^]^ This allows us to hypothesize that a change in the radioresistance of the co‐cultured U251 cells could be a function of its changing stemness characteristics, which is mediated by the endothelial cells, and the presence of the preferential micro‐vessels‐like structures. It is also worth mentioning that CD133 as a stemness marker is sometimes questioned due to its low expression in fresh patient‐derived material,^[^ ^84^, ^85^ ^]^ and in cell lines (such as U251 which shows a CD133+ population of 5%^[^ ^86^ ^]^). CD133‐positive cells are considered to have tumor‐forming properties but it has been reported that also CD133‐negative cells can form tumors.^[^ ^82^, ^87^ ^]^ Many putative markers of GSCs suffer from these cells’ inherent plasticity and from their ability to change from one form to another.^[^ ^27^ ^]^ The presence of endothelial cells in vivo has been shown to promote tumor progression and invasiveness.^[^ ^27^ ^]^ Jamal et al. demonstrated that orthotopic xenografts of GBM cells in mice showed much higher radioresistance when exposed to radiation as compared to the cells in vitro (2D assays). They also show that in the presence of in vivo growth conditions, the cells that exhibit CD133 are less susceptible to double‐strand breakage induction.^[^ ^78^ ^]^ The in vivo environment therefore crucially contributes to the increase in the proportion of cells expressing these stemness markers.^[^ ^78^, ^88^ ^]^ The CD133+ cells are also shown to increase the radioresistance of tumors, and the fraction of cells expressing these markers increases after irradiation.^[^ ^75^ ^]^ Although CD133 can be difficult to isolate and identify in glioma cells in vitro due to cells’ inherent plasticity, the results of our work indicate that the reduction of DNA damage foci and the increase in resistance to proton radiation can be explained by a possible increase in stemness expression, which is mediated by the microenvironment. In such a context, it is also important to mention that our 3D model is still far from a real brain microvessels network. Previous works showed how microfluidic systems combined with ECM‐like matrices, favored the formation of vascularized 3D microtumors models^[^ ^89^ ^]^ (non‐GBM). On the other hand, these materials suffer from well‐known batch‐to‐batch variability,^[^ ^90^, ^91^, ^92^, ^93^ ^]^ which is a detrimental factor for assessing reliably the efficacy of a given treatment. The integration of a microfluidic system would therefore add perfusion features to our reproducible, standardized, and biomimetic 3D in vitro GBM/endothelial model, enabling even higher physiological relevance for proton radiobiology and for the comparison of (patho)physiological mechanisms in glioblastoma therapy.

## 3 Conclusion

In this work, we created micro‐vessels‐like scaffold designs, inspired by brain microvasculature, for studying the response of GBM/HUVEC co‐cultures to proton radiation. The use of beam diameters, shapes, and lengths in the relevant physiological range along with the lattice‐like structure, approaches the vascular environment around which GBM cells migrate and proliferate.^[^ ^26^ ^]^ The use of 2PP allowed to have submicrometric precision in the design with a high rate of reproducibility for accurate comparisons. Further, the presence of HUVECs provided relevant biochemical cues to the environment in which GBM cells grew. The morphologies of cells grown in 3D showed in vivo‐like morphologies in terms of processes/extensions and cytoskeletal area when compared to cells grown on 2D pedestals. In terms of proton radiation response, we observed a clear difference between the co‐culture and monoculture samples, and a difference between cells grown in 2D and 3D, with the latter ones featuring overall a lower amount of DNA damage. The GBM/HUVEC co‐culture samples showed on average a lower amount of DNA damage foci, which can be related to the increasing stemness characteristics observed with GBM cells in co‐culture with HUVECs.^[^ ^80^ ^]^ Our biofidelic 3D GBM model provides a tool for in vitro proton radiobiology studies, which we envision enriching in future with the use of other cell types (e.g., patient‐derived GBM material, pericytes, microglia) and of ultrafast FLASH irradiation modalities.

## 4 Experimental Section

### Two‐Photon Polymerization Setup Configuration

A commercial 2PP setup (Nanoscribe Photonic Professional GT+) was employed to manufacture both the 2D pedestals and 3D scaffolds. The 3D scaffolds were first designed by the Computer‐Aided Design (CAD) software Autodesk Fusion 360 and then exported to an STL format. The STL file was then elaborated by the Nanoscribe DeScribe software to convert it into Nanoscribe's General Writing Language (GWL). The GWL file was finally provided to the NanoWrite program that controls the 2PP setup. During the conversion process, the STL file was sliced into 2D layers and each of these planes was then transformed into a set of hatched lines. A droplet of commercially available negative tone photoresist, known as IP‐Visio (featuring a methacrylate functional group), was cast on cleaned and silanized ITO‐coated (indium‐tin oxide: thickness 18 ±5 nm) soda lime glass substrates (25 × 25 mm, 0.7 mm thickness). The substrates were cleaned and activated with O_2_ plasma (Diener Femto plasma etcher) at a power of 80 W for 10 min, O_2_ flow at 5 sccm, and pressure of 0.1 bar. Consequently, they were silanized by immersion for 1 h in a 2% v/v 3‐(trimethoxysilyl) propyl methacrylate (MAPTMS, Sigma Aldrich)/ethanol solution, then rinsed with acetone and water, and dried with a compressed air gun in between rinsing steps. Silanization increased the adhesion of the photosensitive biomaterial with the substrate. The resin was then exposed to a 780 nm wavelength, femtosecond pulsed laser (100 fs, 50 mW corresponding to 100% power intensity) within the Nanoscribe Photonic Professional GT+ two‐photon polymerization system through a 25× immersion objective (NA = 0.8) and using a “Galvo” configuration where mirrors scanned the laser beam laterally, and the vertical movement was carried out with piezo actuators. The specific parameters used for printing the free‐standing 3D vessel‐like structures were 100% laser power and a scanning speed of 15 mm s^−1^. Concerning the 2D pedestals, the employed parameters were 80% laser power with a 50 mm s^−1^ scanning speed. The slicing (distance between adjacent layers) and hatching (lateral distance between adjacent lines) parameters for both 2D and 3D structures, were set at 800 and 500 nm respectively. Each 3D scaffold and 2D pedestal were fabricated in 7 and 10 min, respectively. Overall, each 2PP printed sample set consisted of 12 scaffolds and three pedestals (≈120 min printing time, Figure S2, Supporting Information). The samples were then chemically developed in propylene glycol monomethyl ether acetate (PGMEA, Sigma‐Aldrich) for 25 min, rinsed with 2‐propanol (IPA, Sigma‐Aldrich) for 5 min, and air‐dried under a chemical fume hood.

### GBM Monoculture and GBM/HUVECs Co‐Culture

Adhesive press‐to‐seal silicone isolator rings (JTR8R‐A‐1.0, Grace Bio‐labs) were used to create a region of 8 mm diameter for the cell culture around the printed scaffolds (Figure S6, Supporting Information). The IP‐Visio structures were then transferred to sterile 60 mm diameter Petri dishes and sterilized by immersion in 70% ethanol for 10 min, washed twice with phosphate‐buffered saline (PBS), and allowed to air‐dry in the sterile cell culture cabinet. The two cell types employed in this study were i) U251 wild‐type high‐grade glioma cell line, and ii) HUVECs. The human GBM U251 (U251 MG, previously known as U373 MG, ATCC HTB‐17) was kindly donated by Prof. Janusz Rak, McGill University, Canada. U251 cells were cultured using Dulbecco's modified Eagle's medium (DMEM, Gibco) with 1% l‐glutamine (Sigma‐Aldrich), 1% penicillin–streptomycin (P/S, Gibco), and 10% fetal bovine serum (FBS, PAN Biotech). DNA profiling using short tandem repeat markers was performed to confirm the origin of U251, and mycoplasma testing was performed monthly using MycoAlertTM Mycoplasma Detection Kit (Lonza). Primary HUVECs were anonymized and isolated from newborn's umbilical cords at the Leiden University Medical Center under the parents’ written informed consent and the Medical Research Ethics Committee (MREC) Leiden, Den Haag, Delft license number B19.026. The employed protocol was an adaptation of the method developed by Jaffe et al. 1973.^[^ ^94^ ^]^ HUVECs were cultured in Endothelial Cell Growth Medium 2 (C‐22011, EGM2, PromoCell). After trypsinization, the HUVECs dilution was made to 600 000 cells per mL and cultured onto the scaffolds in 75 µL droplets for 1 h to enable cell adhesion in a humidified incubator at 37 °C and 5% CO_2_. The cells were then supplemented with 5 mL of EGM2 for each dish and returned to the humidified incubator for 3 days (≈72 h). Afterward, the medium was removed from the dish and U251 wild‐type cells were added, at a concentration of 15 000 cells per mL onto the scaffolds using EGM2 droplets of 75 µL for 1 h to enable adhesion onto the HUVECs (U251:HUVECs ratio of 1:40), before adding 5 mL of EGM2 per dish.^[^ ^80^ ^]^ The GBM monoculture samples were also prepared with an initial density of 15 000 cells per mL onto the scaffolds and also cultured with EGM2. After the addition of GBM cells, the co‐culture samples were kept in culture for 3 days, along with mono‐culture samples before being exposed to proton beam irradiation.

### Scanning Electron Microscopy (SEM)

To prepare the sample for SEM characterization, cells were rinsed with PBS and incubated in a 4% glutaraldehyde (in PBS) solution for 4 h at room temperature. The glutaraldehyde was then removed, and cells were rinsed with PBS. Cells were then dehydrated using 50%, 70%, 90%, and 100% ethanol for 4 min each and then immersed in 50% and 100% solutions of hexamethyldisilazane (HMDS, Sigma‐Aldrich) in 100% ethanol for 15 min each. Finally, the residual HMDS was allowed to evaporate overnight. The whole protocol was carried out under a chemical fume hood.

### Immunofluorescence Staining and Confocal Imaging Configuration

To perform immunofluorescence staining for confocal microscopy, cells were fixed with 4% paraformaldehyde in PBS for 15 min, permeabilized in 0.2% Triton X‐100 for 15 min, and non‐specific protein binding sites were blocked with 5% BSA. Cells were incubated with the DNA damage antibody anti‐gamma H2A.X (phospho serine 139, 1:250 dilution in 1% BSA/PBS, Abcam, ab81299, [EP854(2)Y]) overnight at 4 °C to detect DNA damage, followed by incubation with an Alexa Fluor 647 conjugated Goat Anti‐rabbit IgG secondary antibody (1:500 dilution in 1%BSA, Sigma Aldrich), Phalloidin‐TRITC conjugate (1:100 dilution in 1% BSA, Sigma Aldrich), and FITC‐conjugated sheep anti‐human anti‐von Willebrand factor (vWF, 1:100 dilution in 1% BSA, Abcam) for 60 min at room temperature in a humid chamber. The nuclei were stained with Hoechst 33 258 (1:1000 dilution, Molecular Probes) for 5 min. After the staining, cells were imaged or stored in PBS at 4 °C. Confocal imaging experiments were performed using an upright Zeiss LSM 710 NLO confocal microscope (Carl Zeiss). The 405, 488, 561 and 633 nm laser excitation wavelengths were used for the experiments. 20× (NA = 1.0), and 63× (NA = 1.0) W‐Plan Apochromat water immersion objectives were used to acquire the 2D images and 3D *z*‐stacks. An automatic *z*‐compensation of the laser power was applied to have homogeneous imaging of the sections of the 3D scaffold at different heights. The samples were immersed in PBS at room temperature for the whole duration of the experiments. The images were recorded using Zen (Zeiss) software. The maximum intensity *Z*‐projections and 2D image visualization were performed using Fiji^[^ ^95^ ^]^ while Imaris (Oxford Instruments, UK) was employed for 3D image reconstruction and foci counting using the “cell and vesicle” model (Figure S5, Supporting Information).

### Proton Radiation Experiments Configuration

The layout of the experimental set‐up and the configuration of the R&D beamline of the Holland Proton Therapy Center (HollandPTC) are reported in our previous publication.^[^ ^12^ ^]^ The proton radiation experiments were carried out in the SOBP region of the proton beam dose‐deposition curve. The Bragg Peak was where the maximum proton beam energy was deposited and the SOBP was achieved using a 2D energy modulator which created a plateau characterized by a maximum dose with a uniformity of 98%. A SOBP with a width of 2.5 cm was achieved during the experiments. The beam was passively scattered to produce a large field of 100 × 100 mm with a dose uniformity of 98%. Water equivalent material (Goettingen White Water‐RW3 material, PTW GmbH) was used to adjust the depth so that the sample was located in the middle of the SOBP. The R&D proton beam line of HollandPTC was a fixed horizontal one, therefore the samples had to be held vertically in order to guarantee the dose uniformity during irradiation. For this purpose, specific Petri dish holders were 3D printed by the DEMO (Dienst Elektronische en Mechanische Ontwikkeling) Department at TU Delft. The experiments were conducted with a dose of 8 Gy (delivered with a dose rate of 9 Gy min^−1^) since this dose is relevant in an R&D set‐up to visualize and quantify damage to the DNA in these cells. Large doses of radiation (≈60 Gy) are indeed fractionated to smaller doses for patients and 8 Gy is within the range of clinically relevant doses. With these factors in mind, 8 Gy was chosen as the reference dose for this study. Proton experiments were repeated three times involving a total of eight sample sets per experiment.

### Statistical Analysis

The data were analyzed using GraphPad Prism 9.5.1. A one‐way analysis of variance (ANOVA) test was used to determine the statistical significance. Šidák's multiple comparisons test was performed with a single pooled variance for comparing the multiple data sets. The *p*‐values related to the statistical significance and the sample size (*n*) for each test are displayed in the related figure legends.

### Ethics Approval Statement

The use of patient tissue was approved by the Medical Research Ethics Committee (MREC) Leiden, Den Haag, Delft, license number B19.026.

### Patient Consent Statement

HUVECs were anonymized and isolated from the newborn's umbilical cords under the parents’ written informed consent.

## Conflict of Interest

The authors declare no conflict of interest.

## Supporting information

Supporting Information

## Data Availability

The data that support the findings of this study are available from the corresponding author upon reasonable request.
