# Supplementary material for: Micro‐Vessels‐Like 3D Scaffolds for Studying the Proton Radiobiology of Glioblastoma‐Endothelial Cells Co‐Culture Models
Source: Adv Healthc Mater. 2023 Nov 27;13(6):2302988. doi: 10.1002/adhm.202302988 (PMC11468971; doi:10.1002/adhm.202302988)
Supplement: Supplementary file 1 — Supporting Information [file ADHM-13-2302988-s001.pdf]

ADVANCED HEALTHCARE MATERIALS

Supporting Information

for *Adv. Healthcare Mater.*, DOI 10.1002/adhm.202302988

Micro-Vessels-Like 3D Scaffolds for Studying the Proton Radiobiology of Glioblastoma-Endothelial Cells Co-Culture Models

*Qais Akolawala, Floor Keuning, Marta Rovituso, Wouter van Burik, Ernst van der Wal, Henri H. Versteeg, Araci M. R. Rondon and Angelo Accardo**

Supporting information

Micro-vessels-like 3D Scaffolds for studying the Proton Radiobiology of Glioblastoma-Endothelial Cells Co-culture models

Qais Akolawala[a,b], Floor Keuning[c], Marta Rovituso[b], Wouter van Burik[b], Ernst van der Wal[b], Henri H. Versteeg[d], Araci M.R. Rondon[d], Angelo Accardo[a,*]

[a]Department of Precision and Microsystems Engineering, Faculty of Mechanical, Maritime and Materials Engineering, Delft University of Technology, Mekelweg 2, 2628 CD Delft, The Netherlands.

[b]Holland Proton Therapy Center (HollandPTC), Huismansingel 4, 2629 JH Delft, The Netherlands.

[c]Erasmus Univerisity College, Rotterdam, Nieuwemarkt 1A, 3011 HP Rotterdam, The Netherlands.

[d]Einthoven Laboratory for Vascular and Regenerative Medicine, Division of Thrombosis and Hemostasis, Department of Internal Medicine, Leiden University Medical Center, Albinusdreef 2, 2333 ZA Leiden, The Netherlands

**Corresponding author's e-mail address: A.Accardo@tudelft.nl*

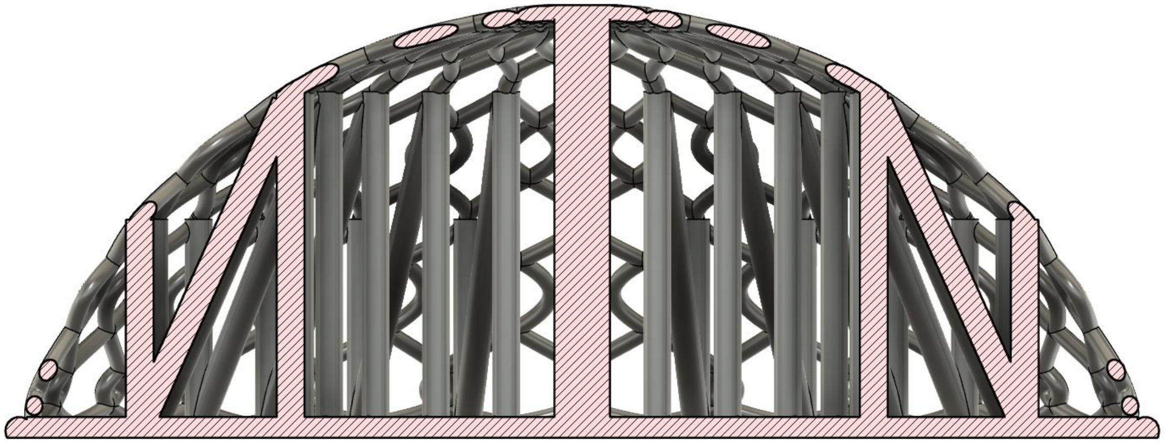

Figure S1. Cross sectional view of the 3D scaffolds. The pink hatched part shows some of the internal supporting pillars.

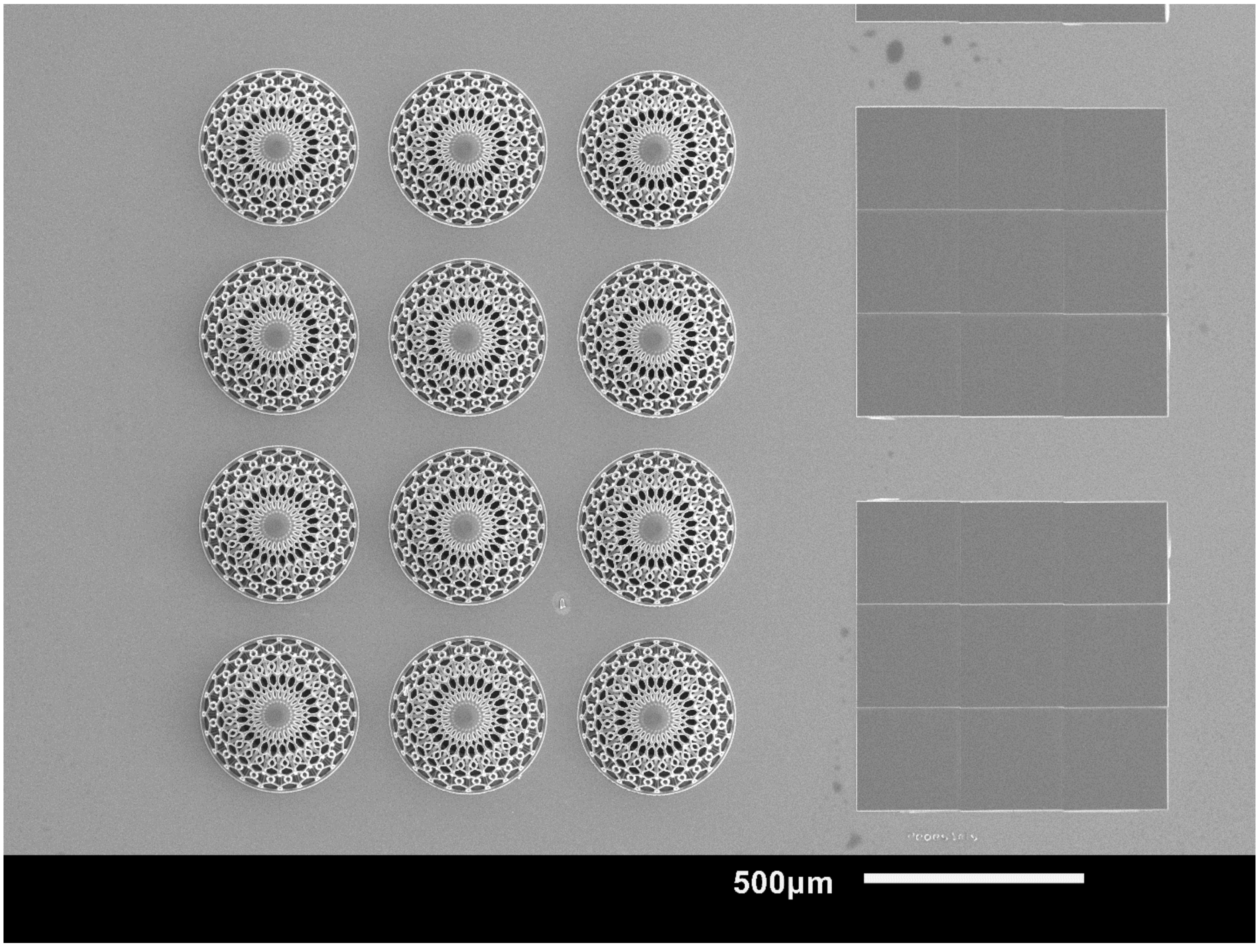

Figure S2. SEM micrograph of 12 scaffolds printed alongside 3 pedestals of 1 mm x 1 mm each (only 2 visible here).

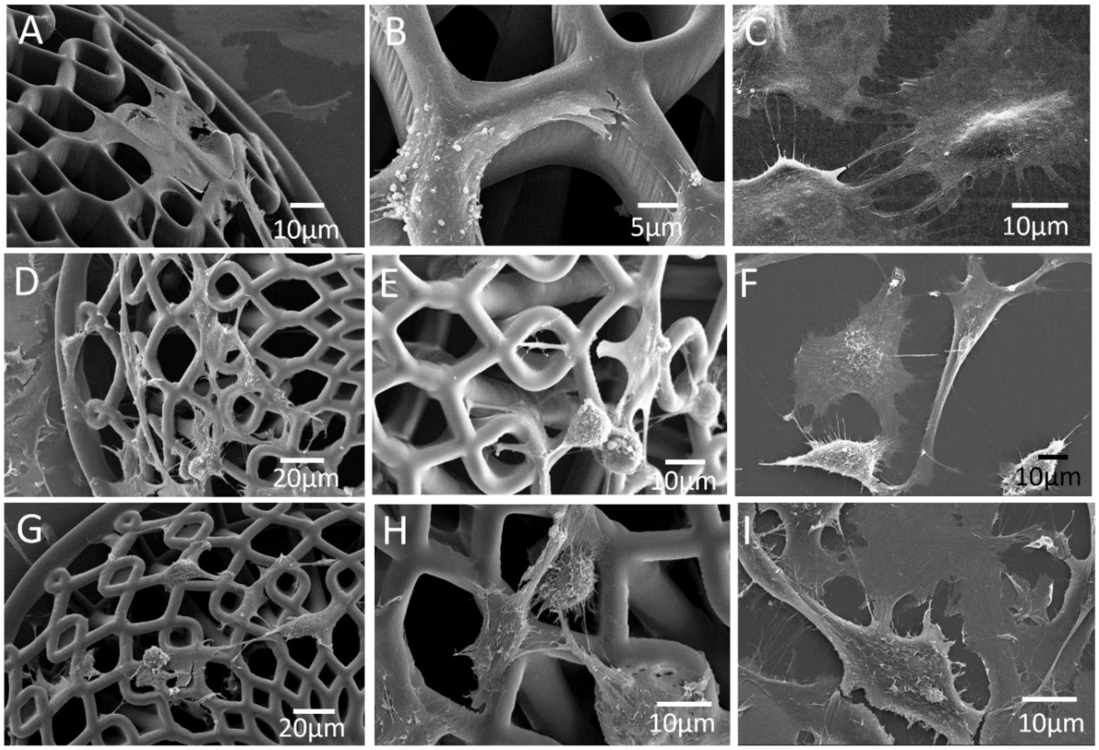

Figure S3. A-C: Low density cell-culture conditions, HUVECs only (Cell density 50,000 cells/mL). D-F: Low density cell-culture conditions GBM only (10,000 cells/mL). G-I: Low density cell-culture conditions, Co-Culture samples.

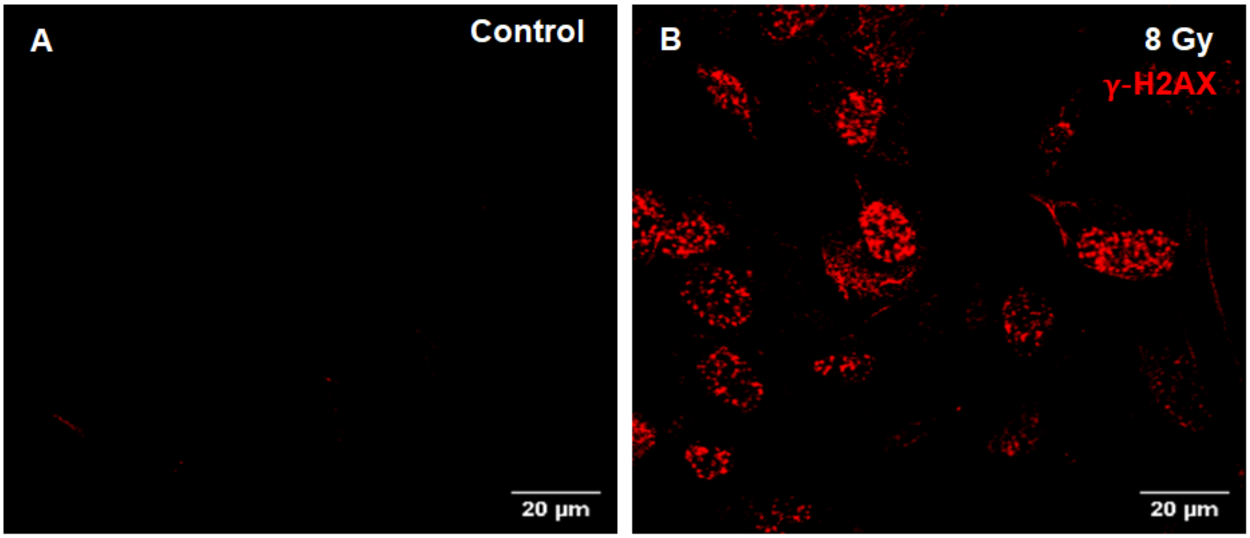

Figure S4. Gamma H2A.X foci formation in U251 cells. (A) Untreated control sample showing no foci. (B) Cells exposed to 8 Gy radiation showing formation of Gamma H2A.X foci within the nuclear region of the cell.

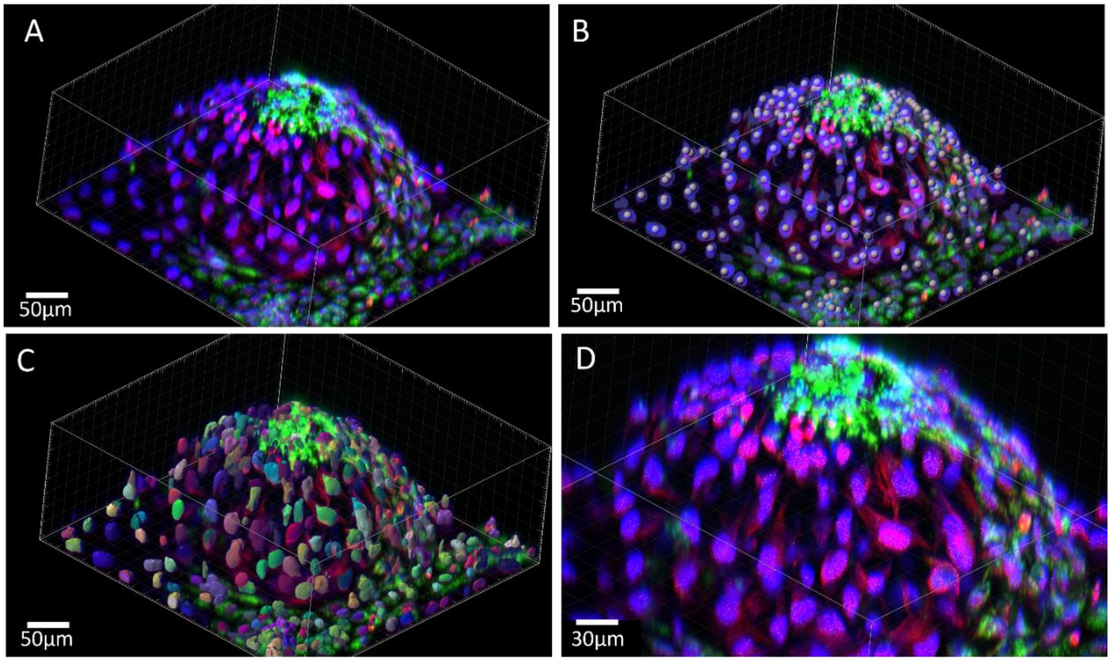

Figure S5. Foci counting strategy, using the Cell and vesicle model of Imaris. (A) 3D reconstruction of the 3D scaffold in Imaris. (B) The nuclei are defined using spots. (C) The nuclei are rendered based on the confocal images of the cells, and the channels are selected. Cells positive for vWF-FITC (Green) are manually excluded. (D) Gamma H2A.X foci (Alexa 647, Red) are generated using the "vesicles" tool. The count of the number of vesicles per nucleus is then exported from Imaris and analysed.

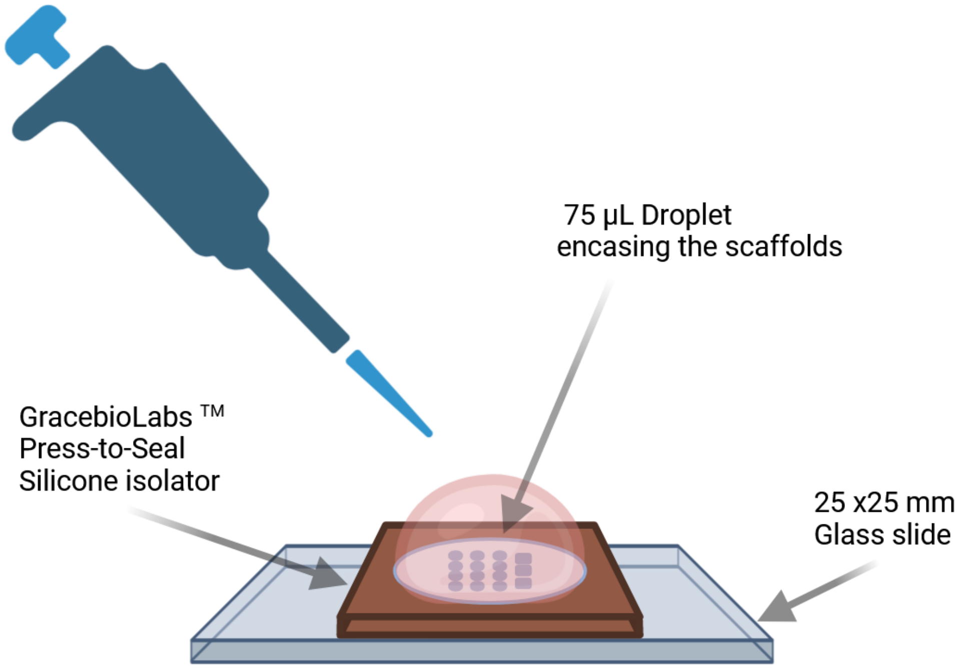

Figure S6. Schematic diagram of the press-to-seal Silicon isolator set-up
